# Comparison of Different Categories of Slovak Tokaj Wines in Terms of Profiles of Volatile Organic Compounds

**DOI:** 10.3390/molecules25030669

**Published:** 2020-02-04

**Authors:** Katarína Furdíková, Andrea Machyňáková, Tereza Drtilová, Ivan Špánik

**Affiliations:** 1 Institute of Biotechnology, Slovak University of Technology in Bratislava, Radlinského 9, 81237 Bratislava, Slovakia; 2 Institute of Analytical Chemistry, Slovak University of Technology in Bratislava, Radlinského 9, 81237 Bratislava, Slovakia; andrea.machynakova@stuba.sk

**Keywords:** Tokaj, comprehensive gas chromatography, high-resolution mass spectrometry, winemaking technologies

## Abstract

The present work deals with the characterization of volatile organic compounds (VOCs) in wines from the Slovak Tokaj wine region. Studied wine samples were divided into three groups—varietal wines from registered Tokaj vine varieties, film wines Tokajské samorodné dry, and naturally sweet botrytized wines Tokaj selections. The VOCs from wines were extracted using optimized solid phase microextraction (SPME) and analyzed by comprehensive two-dimensional gas chromatography (GC×GC) coupled to high-resolution time-of-flight mass spectrometry (HRTOF-MS). In total, 176 VOCs were identified in all 46 studied samples. It was found that the total number of VOCs in varietal wines was generally higher than in botrytized wines. All three studied categories showed characteristic VOC profiles with significant differences. Varietal wines were characterized by higher concentrations of esters and terpenoids originating from grapes. The presence of γ-octalactone, (E)-6-methylhept-2-en-4-one, and lack of benzaldehyde were typical for Tokajské samorodné dry. Tokaj selections expressed the highest concentration of diethyl malate, benzaldehyde, and furfurals. Several interesting trends were also observed. The concentration of fermentation products was highest in varietal wines, while long-term matured Tokaj special wines were typified by the presence of compounds related to noble-rotten raisins (2-phenylacetaldehyde, ethyl 2-phenylacetate, and 2-phenylethanol), wood (*cis*-whisky lactone), and aging (1,1,6-trimethyl-2H-naphthalene, furfural, and 5-methylfurfural).

## 1. Introduction

Tokaj wines belong to the one of the most valued wines with a protected designation of origin in Europe. They originate from the Tokaj wine region, which falls under two adjacent central European countries—Hungary (5800 ha) and Slovakia (929 ha) [1]. The specific microclimate and soil conditions support development of noble-rotten grape berries that are prerequisites for the production of Tokaj wines and are strictly regulated by European as well as national legislation [2]. Accordingly, only three registered grapevine varieties, Furmint, Lipovina, and aromatic Muscat Lunel, can be used to produce Tokaj wines in the Slovak part of Tokaj region. Generally, production is divided into two segments—production of natural grape wines (varietal as well as cuvée) and production of special wines which include Tokajské samorodné (dry or sweet), Tokajský výber (Tokaj selection), Tokajská výberová esencia (Tokaj selection essence), Tokajská esencia (Tokaj essence), Másláš, and Forditáš. Varietal wines, containing at least 85% of wine of one variety, are produced both dry as well as containing residual sugars and are usually not matured in wood barrels. Special wines from Tokaj include the categories “film wines” and “sweet wines with residual sugar derived from grapes” [3], and their production is one of the most laborious. Production of Tokaj special wines always requires noble-rotten raisins—grape berries infected by noble rot caused by *Botrytis cinerea*. Noble-rotten raisin, compared to healthy (uninfected) grape berry, differs in visual appearance as well as in chemical composition. Noble botrytization causes a relevant increase of sugars in berry accompanied with an increase of glycerol, ethanol, citric, gluconic, and succinic acid concentration [4]. Significant changes in volatile organic compound profiles were observed as well. The most important changes were observed in terms of increasing number and concentration of esters, furans, and lactones. Accumulation of volatile acids, higher alcohols, and changes in profiles of terpenoids were also described [4,5].

Harvest and quality of noble-rotten berries are strictly influenced by weather conditions during the vintage. In unfavorable vintages with a low crop of raisins, only dry Tokajské samorodné is produced. Grapes with a concentration of sugars more than 210 g·L^−1^ containing the minimum of raisins are oxidatively macerated up to 24 h and after pressing, grapes must undergo the fermentation process. After the alcoholic fermentation, young wine is transferred into traditional oak barrels with a volume of 136 L, where the process of maturation proceeds. During oxidative aging in barrels, which must last at least one year, a thin biological film is formed on the wine surface. Compared to the biological layer of Jerez flor-wines, the film of yeast in Tokaj wines differs in appearance and, of course, in yeast species representation. Tokaj yeast film is characterized by a lack of *Saccharomyces chevalieri*, *Saccharomyces cheresiensis*, and *Zygosaccharomyces rouxii* [6]. On the contrary, it is formed by pseudomyceliar cells of *Candida stellata* and *Candida zemplinina* [7]. Final Tokajské samorodné dry wine contains up to 10 g·L^−1^ of reducing sugars and approximately 12% *v*/*v* of ethanol [8].

Vintages rich in noble-rotted raisins allow production of naturally sweet wines, where Tokaj selections have the dominant position. The first step in production of these unique wines is manual selection and separation of noble-rotten berries from grape bunches. Collected hand-picked noble-rotten raisins are immersed in grape must with a minimal sugar concentration of 210 g·L^−1^. Noble-rotten raisins macerate in grape must from 24 to 48 h and after pressing, alcoholic fermentation proceeds. Oxidative aging takes place in small 136 L oak barrels and lasts generally 3–5 years. To ensure the quality of Tokaj selection wine, the ratio of mixed grape must and raisins is the most important. The technology still uses old measure named “putňa” (tub in Slovak), which represents approximately 20–25 kg of raisins. The number of tubs mixed with grape must into the final volume of 136 L defines the designation of Tokaj selection and consequently its sensory properties. Tokaj selections are produced as “3-, 4-, 5-, and 6-putňový”, they have typical amber-like color, sweet honey-like taste, and very full sweet botrytic aroma. Both Tokajské samorodné and Tokaj selections are produced from mixed out-plantings, i.e., from grapes of all three registered vine varieties in the approximate ratio Furmint:Lipovina:Muscat Lunel 80:15:5.

Tokaj wines, in terms of their profile of volatile organic compounds (VOCs), are still not well-researched. Only a few papers have been published dealing with this very specific theme [5,9,10,11]. Existing articles about botrytized wines are focused mainly on Sauternes wines [12,13,14], which are, however, produced by different technologies and using different vine varieties (Sémillon, Sauvignon blanc, Muscadelle). In our previous work, VOC profiles of the Tokaj selection 5-putňový were characterized by GCxGC-HRTOF-MS using liquid-liquid extraction (LLE) as the sample treatment procedure. More than 800 organic compounds were detected in Slovak naturally sweet Tokaj wines. The most abundant group of identified VOCs were esters (25), followed by furanoids and pyranoids (20), volatile acids (12), terpenoids (8), higher alcohols (7), carbonyls (6), volatile phenols (5), volatile sulphur compounds (4), and pyrroles. No significant differences were observed between various producers. However, obtained results showed that the vintage has a significant effect on the quality as well as on quantity of identified VOCs [15].

The aim of this work was to characterize volatile organic compounds present in three different categories of Slovak Tokaj wines using comprehensive two-dimensional gas chromatography (GC×GC) coupled to high-resolution time-of-flight mass spectrometry (HRTOF-MS). Comparison of comprehensive VOC profiles of varietal wines produced from registered Tokaj vine varieties with film wines Tokajské samorodné dry and naturally sweet botrytized wines of Tokaj selections will extend the knowledge about these unique wines as well as enable the identification of the most important differences between tested groups.

## 2. Results and Discussion

Tokaj wines represent complicated matrices containing large numbers of organic compounds with different polarities and volatility, belonging to various chemical classes and wide concentration ranges (starting from ng L^−1^ to several hundred mg L^−1^). The easiest and most attractive is direct injection where no sample treatment procedure is required. This method is recommended by International Organization of Vine and Wine (OIV) for analysis of volatiles in dry wines [16]. However, direct injection is suitable mostly for analysis of major volatile components. Moreover, in the case of sweet wines, non-volatile compounds, such sugars, proteins, or polyphenols, could contaminate the injection port, destroy the stationary phase, or cause significant interferences. The key step to eliminate matrix influence is the application of effective sample treatment procedures to extract volatile organic compounds from sweet wines. The most frequently used are solid phase microextraction (SPME), stribar sorptive extraction (SBSE), and static (HS) and dynamic headspace (DHS). In the HS mode, the sample is heated in a sealed vial until equilibrium is reached. After that, a part of the gas phase is injected in the GC. The HS method is limited to the extraction and determination of major volatiles such as acetaldehyde, ethanol, ethyl acetate, ethyl esters of carboxylic acids, or linear and branched alcohols, and is not efficient for the determination of trace organic volatiles. The more suitable sample preparation method seems to be DHS, in which volatile compounds in gaseous phases are purged with inert gas and trapped in a porous sorbent layer followed by desorption and injection into GC. The main DHS advantages are easy sample manipulation, low detection limits, and high sensitivity [17]. A special experimental setup in which the sample is incubated at a fixed temperature under stirring and the volatile compounds are evaporated to the headspace phase and extracted by the sorbent coated on silica fibre is called solid phase microextraction. SPME seems to be the most suitable in case of identification of VOC profile and comparison of VOC profiles for various purposes (e.g., development of aroma compounds), but the quantification and calibration of SPME is challenging [18,19]. The comparison of the pros and cons of sample treatment methods used for extraction of volatiles was recently published by Adams et al. [20].

### 2.1. Optimization of SPME Procedure

In order to receive comprehensive VOC profiles, the SPME working conditions were carefully optimized. During optimization, the time and temperature required to reach equilibrium between liquid and gaseous phase, sorption temperature, and extraction time have been studied. The number of extracted compounds, as well as their peak areas, were used as criteria to select the most suitable SPME conditions. The extraction was performed using the most frequently used SPME fibre for the extraction of VOCs from wines—divinylbenzene/carboxene/polydimethylsiloxane. The first studied parameter, temperature, influences the formation of equilibrium between liquid and gaseous phases. The studied temperature interval started from room temperature and ended with a temperature slightly below the boiling point of ethanol, at 75 °C. It was found that increasing the temperature from 30 °C to 60 °C significantly increased peak areas, as well as the number of detected compounds. The further increase in temperature to 70 °C caused only a slight increase in recorded peak areas. Optimization of equilibration time was performed at 5, 10, 15, 20, 30, 45, and 60 min. The best results were observed for an equilibration time of 60 min. The number of detected peaks significantly increased from 192 (for 10 min) to 482 (60 min), however the increase after 30 min was negligible (only 45 compounds, mostly at a very low concentration level). The second two studied parameters influenced the sorption kinetics from the gaseous phase to sorbent material. The optimization was performed under optimal equilibration time and temperature (60 °C and 30 min). The number of extracted compounds, as well as their peak areas, increased with increasing sorption temperature from 30 °C to 60 °C. Further temperature increases to 75 °C caused only small changes in measured peak areas. Thus, the sorption temperature of 60 °C was selected in future experiments in order to prevent possible decomposition of thermolabile compounds. Similarly, those parameters also increased with increasing sorption time from 10 to 30 min. The further increase of sorption time showed various effects, given some compound peak areas increased, but the number of detected peaks decreased. The experiments resulted in the following optimal SPME working conditions—temperature to reach liquid-gas equilibrium was 60 °C, the time to achieve equilibrium between these phases was 30 min, the extraction temperature 60 °C, and the extraction time was 30 min.

### 2.2. VOC Profile

**Figure 1:**
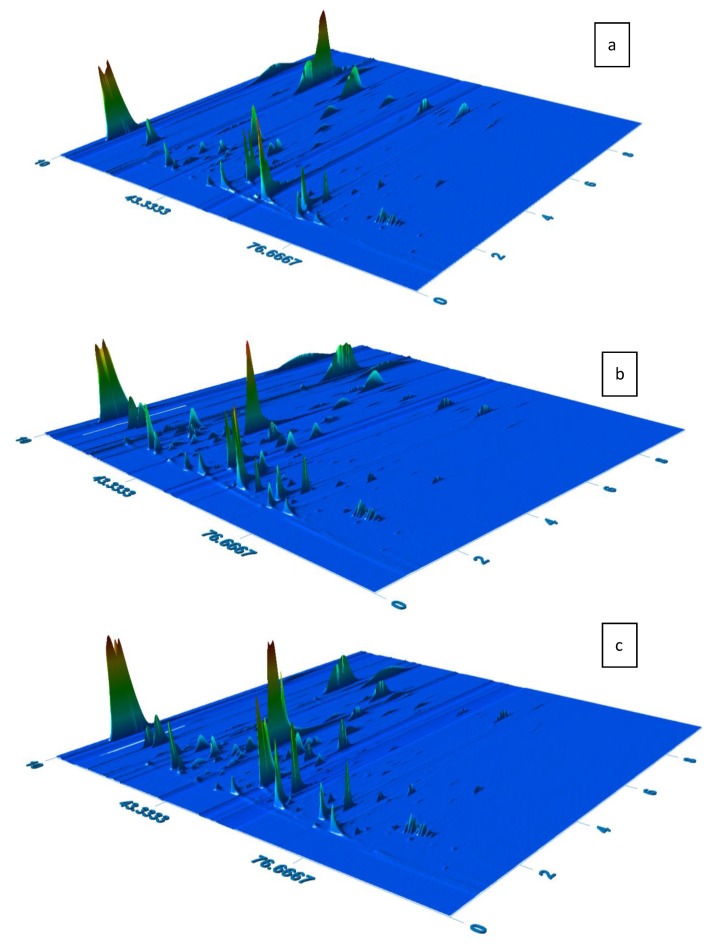
Chromatograms of two-dimensional gas chromatography high-resolution time-of-flight mass spectrometry (GC×GC-HRTOF/MS) of Tokaj wine samples—(**a**) varietal wine Furmint 2015; (**b**) Tokajské samorodné dry 2008; (**c**) 6-putňový Tokaj selection 2003.

In this study, 21 varietal wines produced from Tokaj vine varieties, eight samples of film wines Tokajské samorodné dry, and 17 samples of naturally sweet Tokaj selections were analyzed by HS-SPME-GC×GC-HRTOF/MS to investigate their detailed VOC profiles and to define the most relevant differences between those groups. Representative chromatograms of samples belonging to each group are displayed in Figure 1a–c.

**Figure 2:**
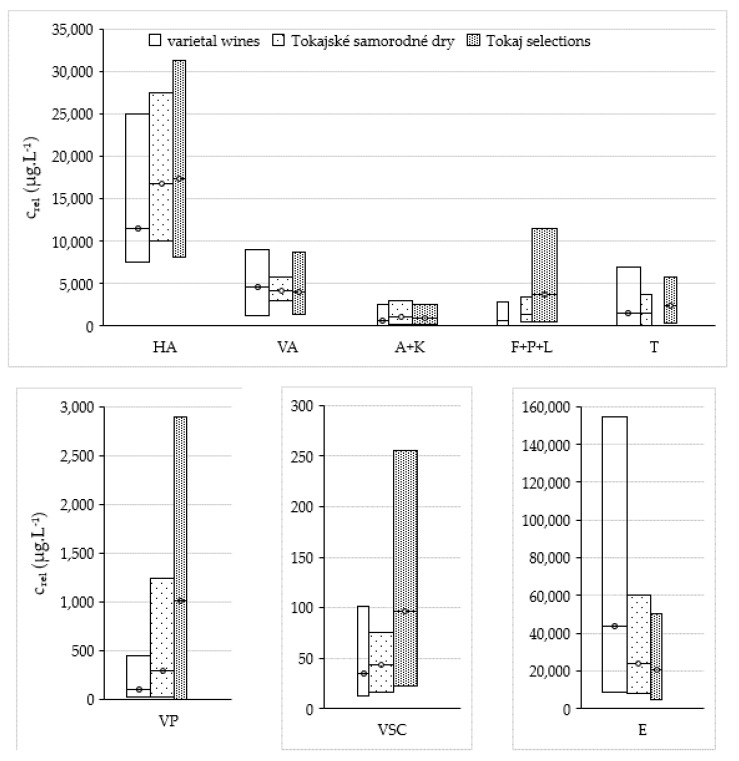
Total concentration of individual groups of volatile organic compounds identified in samples of Tokaj wines. Legend: HA—higher alcohols; VA—volatile acids (except acetic acid); A + K—aldehydes and ketones; F + P + L—furanoids, pyranoids, and lactones; T—terpenoids; VP—volatile phenols; VSC—volatile sulphur compounds; E—esters.

In total, 176 VOCs were identified in all 46 studied samples (74 based on the analysis of standard and 102 tentatively). The list of volatile organic compounds identified in wine samples is shown in Table S1 in the Supplementary Material. It was found that the total number of VOCs in varietal wines was generally higher than in botrytized wines. The number of VOCs in varietal wines produced from Tokaj varieties varied from 45 to 151, in Tokajské samorodné dry from 48 to 141, and in Tokaj selections it was in the range of 38–102. Varietal wines were characterized with a considerably higher number of terpenoids than special wines. Conversely, Tokajské samorodné and Tokaj selections expressed a higher number of furanoid and pyranoid compounds, volatile phenols, and volatile acids. The most abundant groups in investigated wine samples were esters, terpenoids, and higher alcohols and furanoids, which is different to our previous study focused on healthy grape berries or botrytized raisins, where higher alcohols and carbonyls represented the dominant groups of VOC profiles [4,5]. Varietal wines showed a higher total concentration of volatile compounds compared to botrytized ones (average value 62.7 mg·L^−1^ versus 49.4 mg·L^−1^ in Tokajské samorodné and 50.2 mg·L^−1^ in Tokaj selections). The highest differences were recorded in the group of esters—botrytized wines contained on average half the esters of varietal wines, as is shown in Figure 2. Total concentration of higher alcohols, furanoids, pyranoids and lactones, terpenoids, and volatile phenols expressed the opposite trend—their summary concentration was higher in botrytized wines than in varietal ones.

SPME showed a completely different selectivity for particular chemical groups compared to previously used liquid-liquid extraction (LLE) [15]. SPME extracted a significantly higher number of compounds in all chemical classes. The most significant differences in the number of extracted and identified compounds were observed for esters, higher alcohols, terpenoids, and furanoids. SPME extracted 54 esters, while LLE only half of that (25). However, LLE was successful in the extraction of esters with a higher boiling point, e.g., ethyl and diethyl esters of vanillic, succinic, maleic, and levulinic acids. Eighteen higher alcohols were extracted using SPME compared to seven by LLE, but the most abundant (1-hexanol and 2-phenylethanol) were the same in both extraction methods. SPME also extracted higher alcohols with an odd number of carbon atoms (C7 and C9), the presence of which is highly unusual in wine. The significant differences were also observed for the extraction of compounds that are present in grape and noble-rotten berries, such as terpenoids, furans, and lactones. Since those compounds are mostly volatiles, SPME is the preferable method for their extraction. This was also observed for the extraction of those chemical groups from sweet wines. In total, 34 terpenoids and 28 furanoids, pyranoids, and lactones were identified in Tokaj wines using SPME, while only 20 and seven in corresponding chemical classes using LLE. From those observations, it seems that SPME should be the preferable method for the characterization of organic compounds present in wines. SPME also showed completely different selectivity compared to previously used stribars sorptive extraction (SBSE) [21]. Even if sorbent material used was the same (polydimethylsiloxane), SBSE showed better performance in a number of extracted compounds.

In order to consider the influence of noble-rotten raisins and uninfected grape berries on the VOC profile of investigated wines, obtained results were compared to our previous work [4]. Uninfected grape berries and noble-rotten raisins are characterized by higher concentration of C6–C8 alcohols (1-octen-3-ol, 1-octanol, 2-octanol, 3-octanol, 1-hexanol, 2-ethylhexanol, 2-phenylethanol, phenylmethanol) [4,5,9,10,22]. This phenomenon was noticed also in botrytized wines; concentrations of aforementioned alcohols were higher in Tokajské samorodné and Tokaj selections than in varietal wines. The most important was for higher alcohols—2-ethylhexanol, phenylmethanol, and 2-phenylethanol, defined the differences between varietal wines and botrytized wines. Noble-rotten grape berry is characterized by a miscellaneous profile of carbonyl compounds, which are present at relatively high concentrations [4,5]. However, carbonyl profiles of grape must are significantly reduced during the winemaking process. From the total number of dominant C7–C9 carbonyls identified in noble-rotten raisin, only 2-phenylacetaldehyde, benzaldehyde, and 1-phenylethanone remained dominant in Tokaj selections. These compounds were previously confirmed in botrytized wines and considered the main scents of their typical sweet aroma [12]. Tokajské samorodné was characterized by an increased concentration of 2-phenylacetaldehyde, E-6-methylhept-2-en-4-one, and absence of benzaldehyde.

Fresh, healthy grapes contain only trace concentrations of a limited number of esters [23,24], while the ester profile of noble-rotten berry is much more diversified. Dominant esters of noble-rotten raisins are ethyl 2-phenylacetate, 2-phenylethyl acetate, ethyl octanoate, and hexyl acetate [4,5]. Typical fermentative wine esters (ethyl hexanoate, octanoate, and decanoate) were dominant in all studied wine samples but their concentrations in Tokaj special wines were significantly lower compared to varietal ones (probably due to acidic hydrolysis of esters during long-term wine aging). On the other hand, concentrations of sweet-scented esters typical for raisins—ethyl 2-phenylacetate and 2-phenylethyl acetate, and esters typical for long-term maturation in contact with yeast lees—ethyl 2-hydroxypropanoate and diethyl butanedioate—were significantly higher in Tokaj special wines. 

The absolute lack or only the trace concentrations of unbranched C6–C10 volatile acids is typical for healthy grapes [4,23,25]. On the contrary, their concentration in noble-rotten raisin is slightly higher. The concentration of dominant volatile acids produced by the metabolism of yeast during alcoholic fermentation—hexanoic, octanoic, and decanoic acid—were statistically similar in all studied wine samples. The most important volatile acid differentiating Tokaj selection wines was 2-ethylhexanoic acid. 

The concentration of terpenoids in wine is influenced by its varietal composition. The comparison of healthy berry to corresponding noble-rotten raisin of the same vine variety showed that the concentration of identified terpenoids increased due to botrytization [4]. Despite this, the concentration of the most common wine terpenoids (nerol, linalool, hotrienol) in Tokaj special wines was lower than in varietal ones. The decrease and increase of norisoprenoid 1,1,6-trimethyl-2H-naphthalene in special wines probably relate to slow oxidation during long-term aging [26]. 

Noble-rotten raisins and botrytized wines are rich in O-heterocyclic compounds, such as furanoids, pyranoids, and lactones. Dominant furanoid compounds of noble-rotten berry—2-pentylfuran and 5-ethyl-3H-furan-2-one [4]—were not identified in any of the studied samples. Other dominant compounds—γ-nonalactone and γ-butyrolactone—were identified in examined wines but were not responsible for statistically significant differences between samples. Both Tokajské samorodné and Tokaj selections showed high concentrations of *cis*-whiskey lactone—a volatile compound strictly connected with wood [27]. The volatile profile of Tokajské samorodné was characterized by significantly higher concentrations of γ-octalactone and 2-hydroxyglutaric acid γ-lactone. Tokaj selections contained the highest concentrations of furfural and 5-methylfurfural—volatiles typical for old sweet wines (liqueur as well as naturally sweet).

### 2.3. Multivariate Analysis of Tokaj Wines’ Volatile Compounds

**Figure 3:**
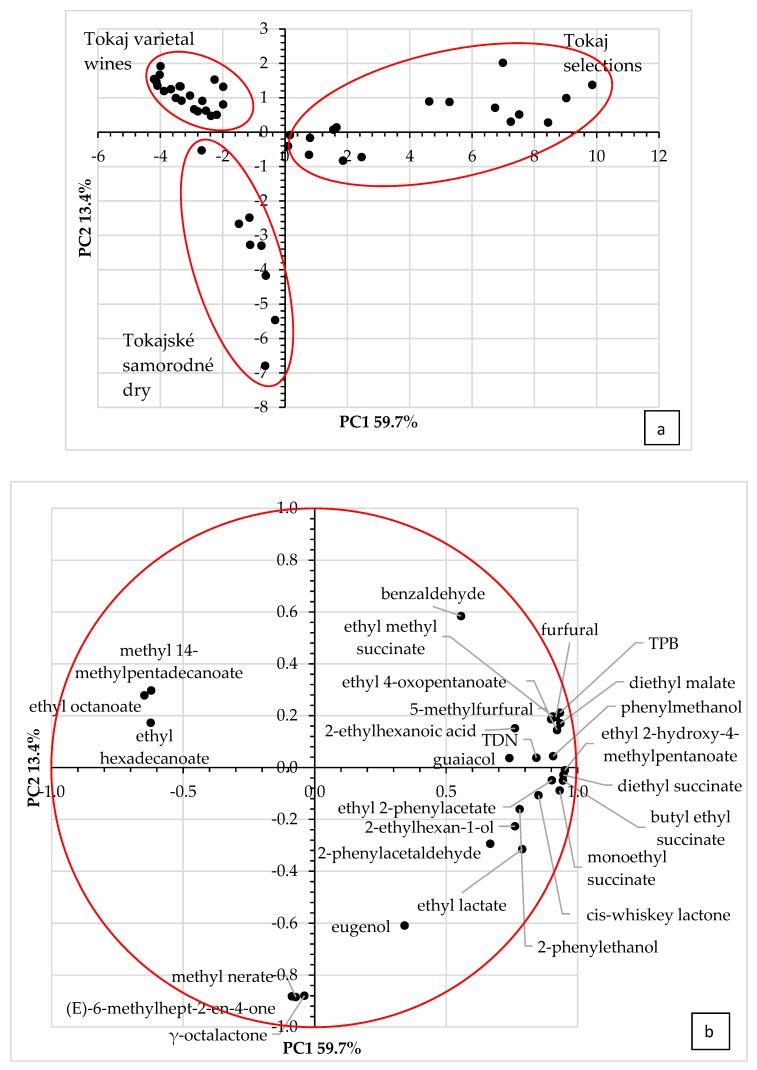
Plot (**a**) and loading plot (**b**) of the first and second principal components (PCs) after PCA of VOC profiles of Tokaj varietal wines, wine classified as Tokajské samorodné dry, and Tokaj selections.

VOCs determined in studied Tokaj wines underwent statistical data treatment using one-way ANalysis Of VAriance (ANOVA) and Principle Component Analysis (PCA). Firstly, *p*-values and Fischer ratios (FR) were calculated by one-way analysis of variance to determine VOCs responsible for the main differences between wines. *p*-Values higher than 0.05 were reported for 64 of 176 total VOCs, meaning that 112 VOCs showed statistically significant differences between three examined groups of wines (Table S1 in Supplementary Material). This number of variables was too large for a brief PCA analysis, so FR values were considered as a sorting key. The higher FR numerical value obtained for compounds represented the greater variance between groups of wines. The correlation matrix used for the PCA was calculated in order to discriminate the variables, followed by selection of 28 VOCs with an FR higher than 16.0 (Table 1). PCA explained 73.1% of total variance—59.7% for PC1 and 13.4% for PC2. As shown in Figure 3a, these PCs could be used to distinguish compared groups of wines. PC1 was responsible for the differentiation of Tokaj selections from other groups of Tokaj wines. Compounds such as methyl 14-methyl pentadecanoate, ethyl octanoate, and ethyl hexadecanoate were negatively correlated with this PC and mostly characterized the group of Tokaj varietal wines (Figure 3b). Ethyl 2-hydroxy-4-methyl pentanoate, diethyl butanedioate, butyl ethyl butanedioate, 4-ethoxy-4-oxobutanoic acid, and ethyl 2-phenylacetate were positively correlated and typical for samples of naturally sweet botrytized Tokaj selections. Varietal wine samples produced from registered Tokaj vine varieties and samples of film wines Tokajské samorodné dry can be distinguished based on PC2, albeit the variance of this factor was much lower than the variance of PC1. Volatile compounds methyl (2Z)-3,7-dimethylocta-2,6-dienoate, (E)-6-methylhept-2-en-4-one, and 5-butyloxolan-2-one correlated negatively with PC2 and separated varietal wines from Tokajské samorodné dry.

## 3. Materials and Methods

### 3.1. Wine Samples

All 46 studied wine samples were obtained from three Slovak wine-making companies, namely Ostrožovič spol. s r.o. (Veľká Tŕňa, Slovakia), Anna Nagyová—ZLATÝ STRAPEC (Viničky, Slovakia), and Tokaj & Co., s.r.o. (Malá Tŕňa, Slovakia). Samples were divided into 3 groups—the first group contained 21 varietal wines, which were produced from registered Tokaj vine varieties (Furmint (8 samples), Lipovina (6), and Muscat Lunel (7)); the second group consisted of 17 Tokaj selections (Tokajský výber 3-putňový (4 samples), Tokajský výber 4-putňový (4), Tokajský výber 5-putňový (4), and Tokajský výber 6-putňový (5); and the third group included 8 samples of Tokajské samorodné dry. The studied wines were of different vintages and sugar-based categories. None of varietal wines had matured in oak barrel. A list of the samples with brief characterization is shown in Table 2.

### 3.2. Analysis of VOC Profiles by GC×GC

Volatile organic compounds were extracted from wine samples using an optimized SPME procedure by MultiPurpose Sampler (Gerstel, Mülheim an der Ruhr, Germany). Six millilitres of wine sample together with 0.5 g NaCl (p.a., Merck, Darmstadt, Germany) and 20 µL of ethanol solution of 1.60 mg·L^−1^ benzophenone (internal standard) was placed into a 20 mL glass headspace vial and sealed with metallic hole cup and PTFE/Silicone septa. The extraction of VOCs was performed by a PDMS/CAR/DVB SPME fibre (50/30 μm thickness) obtained from Supelco (Bellefonte, PA, USA). The conditioning of the fibre prior to use was performed by heating in the needle heater of the autosampler under the conditions recommended by the manufacturer. The adsorption of VOCs from wine samples on the SPME fibre took 30 min at 60 °C while the solution was stirred at 8,3 Hz. Desorption was performed in the GC injector in splitless mode at 220 °C for 2 min.

The analysis of samples was performed using Pegasus GC×GC-HRTOF-MS (Leco Corporation, St. Joseph, MI, USA) consisting of a Agilent 7890B gas chromatograph (Agilent Technologies, Palo Alto, CA, USA), HRTOF-MS (Leco, San Joseph, USA), ZX-2 noncryogenic dual-stage thermal loop modulator and equipped with Gerstel MPS2 autosampler (Gerstel, Mülheim, Germany). The column setup consisted of a 30 m × 0.25 µm × 0.25 µm DB-FFAP column (Agilent Technologies) in the first dimension and a 1.6 m × 0.25 mm × 0.25 µm Rxi-17Sil (Restek, Bellefonte, PA, USA) in the second dimension. Helium (99.996 % purity, Merck) with flowrate 1 mL·min^−1^ was used as a carrier gas. The temperature program started from 40 °C kept for 10 min, with a slow gradient of 2 °C.min^−1^ to a final temperature of 220 °C kept for 5 min. Samples were injected into a splitless injector heated at 220 °C. A modulator was kept at 15 °C higher compared to the actual oven temperature with a modulation period of 10 s. The temperature of the second oven was maintained in a 5 °C offset compared to the first oven temperature program. HRTOF-MS spectra were obtained at an ionization energy of 70 eV, temperature of the ion source was set to 250 °C, and the detector was maintained at 1860 V. The signal acquisition rate was 100 spectra.s^−1^ in the mass range m/z of 29–550. 

The recorded chromatograms were evaluated using LECO ChromaTOF-HRT 1.90.60 Software and the US National Institute of Standards and Technology (NIST14) mass spectra library. The identification of VOC was confirmed by accurate mass measurements and comparison of the measured retention index (RI), with the RI obtained by the injection of authentic standards or with a reference value obtained from [28]. The VOC was considered identified if the difference between the experimental and reference RI was less than 20 units.

For semiquantitation purposes, relative concentrations (crel) of VOCs were calculated by the ratio of each individual peak area to the area of internal standard and converted to concentration equivalents based on the internal mass added [29].

### 3.3. Chemicals

The following authentic standards were used for identification of VOCs by GC×GC—3-methyl-1-butanol, 1-hexanol, 3-hexen-1-ol, 3-octanol, 2-octanol, 1-octen-3-ol, 1-heptanol, 2-ethyl-1-hexanol, 2-nonanol, 1-octanol, 1-nonanol, 1-decanol, phenylmethanol, 2-phenylethanol, 2-nonanone, nonanal, decanal, benzaldehyde, phenylacetaldehyde, 1-phenylethanone, furan-3-carbaldehyde, furan-2-carbaldehyde, ethyl furan-2-carboxylate, 5-methylfuran-2-carbaldehyde, 2H-furan-5-one, 5-butyloxolan-2-one, (4R,5R)-5-butyl-4-methyloxolan-2-one, 5-pentyloxolan-2-one, 5-acetyloxolan-2-one, 5-hexyloxolan-2-one, oxolan-2-one, 5-(hydroxymethyl) furan-2-carbaldehyde, 6-propyloxan-2-one, hexyl acetate, ethyl phenylacetate, methyl phenylacetate, ethyl benzoate, methyl benzoate, ethyl heptanoate, ethyl hexanoate, diethyl succinate, ethyl nonanoate, methyl decanoate, 2-phenylethyl acetate, ethyl hexadecanoate, ethyl octanoate, ethyl decanoate, methyl dodecanoate, ethyl dodecanoate, methyl octanoate, hexanoic acid, heptanoic acid, octanoic acid, nonanoic acid, decanoic acid, dodecanoic acid, hexadecanoic acid, sulcatol, nerol oxide, linalool, 4-terpinenol, hotrienol, myrcenol, citronellyl acetate, α-terpineol, citral, β-citronellol, α-citronellol, β-damascenone, nerol, geranic acid, α-ionene, and benzophenone. All standards had purity higher than 99.5% and were obtained from Merck.

### 3.4. Statistical Analysis

All analyses were performed in triplicate. Results of the GC×GC were analyzed by one-way analysis of variance (ANOVA) using software Statistica 12 (StatSoft, Tulsa, OK, USA). Fisher’s ratios and *p*-values were calculated for three main groups—varietal wines, Tokajské samorodné, and Tokaj selections wines. *p*-Values ≤ 0.05 were considered statistically significant. VOCs with a Fisher ratio higher than 16.0 served as input parameters for principal component analysis (PCA) to graphically illustrate interactions between groups.

## 4. Conclusions

The aim of this work was to characterize and compare the VOC profiles of three different categories of wines produced in the Slovak Tokaj wine region—varietal wines, Tokaj selections and Tokajské samorodné dry—using comprehensive two-dimensional gas chromatography (GC×GC) coupled to high-resolution time-of-flight mass spectrometry (HRTOF-MS). An optimized SPME procedure resulted in a 60 °C equilibration temperature, 30 min equilibration time, 60 °C extraction temperature, and 30 min extraction time as the most suitable extraction conditions in terms of number of extracted compounds and corresponding peak areas. The SPME showed a completely different selectivity for particular chemical groups compared to previously used liquid-liquid extraction. The most significant differences in the number of extracted and identified compounds were observed for esters, higher alcohols, terpenoids, and furanoids. From those observations, it seems that SPME should be the preferable method for the characterization of organic compounds present in wines.

VOC profiles were evaluated by statistical methods ANOVA and PCA. It was found that all three categories of wine were unique, and their VOC profiles expressed significant differences. Varietal wines produced from registered Tokaj vine varieties were characterized by the presence of higher concentration of fermentation esters (ethyl octanoate, ethyl hexadecanoate) and terpenoids originating from grapes. Tokaj special wines generally contained lower concentration of esters, while diethyl succinate, monoethyl succinate, and diethyl malate were dominant. Profiles of grape terpenoids in long-term matured special wines is poorer, with the dominant compounds being methyl nerate and norisoprenoids TPB and TDN. Furthermore, a high concentration of O-heterocyclic compounds was typical for Tokajské samorodné dry and Tokaj selections.

Interestingly, the VOC profile of Tokaj special wines is directly connected to botrytized raisins. Some of them (e.g., 2-ethylhexanol, phenylmethanol, 2-phenylethanol, 2-phenylacetaldehyde) were identified in both raisins and botrytized wines, however, some of dominant constituents of the VOC profile of noble-rotten grape berry vanish or transform during winemaking process.

## Figures and Tables

**Table 1:** Volatile organic compounds responsible for the greatest differences between Tokaj varietal wines, Tokajské samorodné dry, and Tokaj selections.

RI	Compound	FR	Varietal Wines			Tokajské Samorodné Dry			Tokaj Selections		
			Avg	Min	Max	Avg	Min	Max	Avg	Min	Max
1657	methyl (*2Z*)-3,7-dimethylocta-2,6-dienoate (methyl nerate)	54.1	-	nd	nd	110	nd	237	-	nd	nd
1520	(*E*)-6-methylhept-2-en-4-one	46.6	-	nd	nd	295	132	678	-	nd	nd
2450	2-[(*1E*)-1,3-butadien-1-yl]-1,3,4-trimethylbenzene (TPB)	46.1	-	nd	nd	-	nd	nd	246	47	491
1666	diethyl butanedioate (diethyl succinate)	43.0	1006	459	3863	2492	1071	3721	6293	1818	10590
1911	5-butyloxolan-2-one (*γ*-octalactone)	39.7	8	nd	30	199	52	495	13	nd	66
1567	5-methylfuran-2-carbaldehyde (5-methylfurfural)	35.9	7	2	34	30	9	74	271	31	623
2395	4-ethoxy-4-oxobutanoic acid (monoethyl succinate)	33.3	242	82	764	619	268	1272	1182	461	2164
2191	2-methoxy-4-prop-2-enylphenol (eugenol)	30.6	-	nd	nd	26	nd	40	24	nd	56
1860	2-methoxyphenol (guaiacol)	29.6	-	nd	nd	2	nd	5	11	nd	24
1964	(*4R,5R*)-5-butyl-4-methyloxolan-2-one (*cis*-whiskey lactone)	27.2	-	nd	nd	189	nd	495	519	148	1212
1515	ethyl 2-hydroxy-4-methylpentanoate	25.8	41	nd	151	95	59	180	222	87	429
1820	butyl ethyl butanedioate	25.0	31	12	152	161	31	346	384	134	864
1869	phenylmethanol	23.8	14	nd	35	25	16	32	61	14	124
2031	diethyl 2-hydroxybutanedioate (diethyl malate)	22.5	62	24	281	147	34	300	998	nd	2428
2166	methyl 14-methylpentadecanoate	21.7	120	nd	282	49	nd	91	nd	nd	nd
1630	2-phenylacetaldehyde	21.4	89	8	269	208	nd	355	277	162	469
1686	1,1,6-trimethyl-*2H*-naphthalene (TDN)	21.2	141	nd	812	576	nd	1120	1375	217	3081
1420	ethyl octanoate	20.3	13,788	4410	26,523	6219	2342	14,581	3261	743	7355
1567	ethyl 4-oxopentanoate	20.2	1	nd	6	1	nd	5	45	nd	153
1770	ethyl 2-phenylacetate	19.4	266	87	676	683	286	1282	1224	523	2901
1457	furan-2-carbaldehyde (furfural)	18.6	42	nd	291	123	13	199	1208	98	3520
1364	ethyl 2-hydroxypropanoate	18.2	180	nd	1037	1560	397	4151	2043	39	4715
1520	benzaldehyde	17.2	191	101	427	-	nd	nd	291	34	551
1467	2-ethylhexan-1-ol	16.7	68	22	182	180	83	339	261	10	671
2235	ethyl hexadecanoate	16.4	1345	nd	2410	612	nd	1554	187	nd	1532
1631	4-*O*-ethyl 1-*O*-methyl butanedioate (ethyl methyl succinate)	16.2	1	nd	17	5	nd	17	81	nd	203
1935	2-ethylhexanoic acid	16.2	nd	nd	nd	nd	nd	nd	40	nd	110
1896	2-phenylethanol	16.0	2767	1778	5285	4976	3042	7180	6602	2885	14,758

Legend—relative concentrations are expressed as equivalents of benzophenone in µg·L^−1^. RI—experimental value of retention index; avg—average value of relative concentration; min—minimal determined concentration; max—maximal determined concentration; FR—Fischer ratio; nd—not detected; *p* ≤ 0.05.

**Table 2:** List of studied Tokaj wine samples.

Sample		Count	Vintage	Residual Sugars (g·L^−1^)	Alcohol (% *v*/*v*)
Varietal wines	Furmint	8	2013–2016	≤4	11–15
	Lipovina	6	2015–2016		
	Muscat Lunel	7	2013–2016		
Tokajské samorodné dry		8	2008–2016	≤ 10	11–13
Tokaj selections	Tokajský výber 3-putňový	4	2000–2009	60 – 90	10–12
	Tokajský výber 4-putňový	4	2000–2009	90–120	
	Tokajský výber 5-putňový	4	2000–2004	120–150	
	Tokajský výber 6-putňový	5	2002–2011	≥150	

## Supplementary Materials

The following are available online, Table S1: Volatile organic compounds identified in samples of Tokaj varietal wines, Tokajské samorodné dry and Tokaj selections.
