# Supplementary material for: Comparison of Different Categories of Slovak Tokaj Wines in Terms of Profiles of Volatile Organic Compounds
Source: Molecules. 2020 Feb 4;25(3):669. doi: 10.3390/molecules25030669 (PMC7038026; doi:10.3390/molecules25030669)
Supplement: Supplementary file 1 [file molecules-25-00669-s001.pdf]

Table S1 Volatile organic compounds identified in samples of Tokaj varietal wines, Tokajské samorodné dry and Tokaj selections

			Varietal wines			Tokajské samorodné suché			Tokaj selections		
**RI**	**Compound**	**FR**	**avg**	**min**	**max**	**avg**	**min**	**max**	**avg**	**min**	**max**
	Higher alcohols										
1200	2-methylbutan-1-ol	0.2	457	nd	1506	512	nd	1613	561	nd	1489
1210	3-methylbutan-1-ol	6.3*	6048	5073	9136	7930	6216	9650	7017	4415	8730
1348	hexan-1-ol	4.0*	680	418	1236	934	546	1793	973	485	1726
1355	hex-3-en-1-ol	2.9	33	5	224	46	9	85	12	nd	35
1386	octan-3-ol	1.5	15	6	61	32	nd	187	30	4	72
1405	octan-2-ol	2.9	2	nd	19	25	nd	143	11	nd	44
1442	oct-1-en-3-ol	2.3	31	nd	90	87	nd	464	78	nd	249
1449	heptan-1-ol	0.5	48	23	138	58	nd	233	62	17	165
1467	2-ethylhexan-1-ol	16.7*	68	22	182	180	83	339	261	10	671
1510	nonan-2-ol	4.5*	20	nd	53	44	19	121	58	nd	180
1529	butane-2,3-diol	0.7	1097	65	6318	1576	nd	4710	1437	255	2576
1545	octan-1-ol	2.6	75	48	159	119	70	293	93	nd	165
1647	nonan-1-ol	0.2	54	32	149	64	27	186	60	11	122
1682	(*Z*)-non-3-en-1-ol	3.0	8	nd	23	17	nd	71	4	nd	21
1749	decan-1-ol	6.6*	108	29	196	120	54	236	55	nd	163
1784	(*Z*)-dec-4-en-1-ol	3.4*	2	nd	16	5	nd	28	nd	nd	nd
1869	phenylmethanol	23.8*	14	nd	35	25	16	32	61	14	124
1896	2-phenylethanol	16.0*	2767	1778	5285	4976	3042	7180	6602	2885	14758
1940	dodecan-1-ol	0.7	35	nd	97	33	nd	120	24	nd	69
2387	hexadecan-1-ol	2.9	4	nd	26	6	nd	19	nd	nd	nd
	**Volatile acid**s										
1560	2-methylpropanoic acid	8.4*	35	nd	132	104	25	181	51	19	114
1620	butanoic acid	11.5*	nd	nd	nd	nd	nd	nd	37	nd	103
1627	3-methylbutanoic acid	3.4*	25	nd	92	95	nd	250	59	nd	211
1733	pentanoic acid	11.2*	nd	nd	2	nd	nd	nd	3	nd	9
1757	2-methylhexanoic acid	7.5*	nd	nd	nd	nd	nd	nd	67	nd	272
1834	hexanoic acid	0.2	619	27	1608	673	441	1211	587	252	853
1935	2-ethylhexanoic acid	16.2*	nd	nd	nd	nd	nd	nd	40	nd	110
1956	heptanoic acid	1.4	7	nd	50	15	nd	89	17	nd	46
2038	2-ethylheptanoic acid	5.2*	5	nd	24	nd	nd	nd	nd	nd	nd
2046	octanoic acid	2.3	2520	1015	4378	2316	1964	2532	2133	962	3123
2140	nonanoic acid	7.9*	53	nd	124	55	nd	91	117	nd	216
2251	decanoic acid	2.3	1266	175	2135	896	511	1205	837	147	3418
2485	dodecanoic acid	0.8	34	nd	338	17	nd	62	12	nd	36
2560	2-phenylacetic acid	5.7*	2	nd	20	7	nd	23	17	nd	50
2713	tetradecanoic acid	9.9*	2	nd	21	3	nd	26	19	nd	44
2890	hexadecanoic acid	2.3	19	nd	47	20	nd	50	31	nd	58
	**Esters**										
1226	ethyl hexanoate	14.6*	4186	2041	10571	2870	1351	8104	990	128	2552
1270	hexyl acetate	3.0	624	nd	5527	nd	nd	nd	nd	nd	nd
1305	ethyl hex-2-enoate	4.0*	13	nd	50	38	nd	202	nd	nd	nd
1337	ethyl heptanoate	4.2*	118	nd	371	87	nd	589	11	nd	48
1364	ethyl 2-hydroxypropanoate	18.2*	180	nd	1037	1560	397	4151	2043	39	4715
1388	methyl octanoate	3.1	396	nd	2295	114	nd	520	138	nd	550
1420	ethyl octanoate	20.2*	13788	4410	26523	6219	2342	14581	3261	743	7355
1450	3-methylbutyl hexanoate	11.6*	228	nd	726	78	nd	319	13	nd	68
1515	ethyl 2-hydroxy-4-methylpentanoate	25.8*	41	nd	151	95	59	180	222	87	429
1530	propyl octanoate	7.6*	18	nd	71	nd	nd	nd	nd	nd	nd
1537	2-methylpropyl octanoate	11.9*	145	nd	394	56	nd	193	8	nd	34
1541	ethyl nonanoate	3.2*	265	77	1034	144	36	579	125	nd	406
1543	diethyl propanedioate	14.9*	3	2	21	6	3	14	30	nd	93
1567	ethyl 4-oxopentanoate	20.2*	1	nd	6	1	nd	5	45	nd	153
1572	3-methylbutyl 2-hydroxypropanoate	13.1*	13	nd	78	174	39	482	116	nd	345
1580	methyl decanoate	3.1	814	55	5220	245	nd	1329	164	nd	1654
1600	methyl benzoate	3.4*	5	nd	45	24	nd	51	12	nd	49
1606	diethyl 2-methylbutanedioate	11.5*	nd	nd	nd	2	nd	10	82	nd	281
1631	4-*O*-ethyl 1-*O*-methyl butanedioate	16.2*	1	nd	17	5	nd	17	81	nd	203
1633	ethyl decanoate	6.9*	13120	1113	55527	3955	1563	9429	1545	333	4642
1645	3-methylbutyl octanoate	10.5*	1129	181	3327	456	184	890	84	nd	366
1652	ethyl benzoate	6.5*	23	nd	81	35	nd	134	101	19	381
1666	diethyl butanedioate	43.0*	1006	459	3863	2492	1071	3721	6293	1818	10590
1675	ethyl dec-9-enoate	5.5*	225	20	1106	69	14	263	24	nd	66
1720	methyl 2-phenylacetate	7.1*	nd	nd	nd	nd	nd	nd	3	nd	11
1720	propyl decanoate	2.8	24	nd	184	nd	nd	3	nd	nd	nd
1734	ethyl undecanoate	3.7*	41	nd	210	33	nd	156	2	nd	22
1750	2-phenylethyl formate	3.0	nd	nd	5	6	nd	35	1	nd	11
1750	2-methylpropyl decanoate	1.9	83	nd	608	7	nd	24	29	nd	122

RI	Compound	FR	Varietal wines avg	min	max	Tokajské samorodné suché avg	min	max	Tokaj selections avg	min	max
1755	methyl 2-hydroxybenzoate	10.4*	5	nd	32	8	nd	39	26	nd	63
1770	ethyl 2-phenylacetate	19.3*	266	87	676	683	286	1282	1224	523	2901
1780	diethyl pentanedioate	12.9*	7	nd	31	25	13	44	44	nd	105
1785	methyl dodecanoate	2.8	115	nd	862	60	nd	392	nd	nd	nd
1791	ethyl 2-hydroxybenzoate	11.4*	nd	nd	nd	3	nd	17	17	nd	53
1799	2-phenylethyl acetate	2.7	571	143	1604	869	407	1287	801	246	2040
1820	butyl ethyl butanedioate	25.0*	31	12	152	161	31	346	384	134	864
1825	ethyl dodecanoate	2.5	2166	65	17109	724	nd	2665	79	nd	296
1864	3-methylbutyl decanoate	4.8*	787	23	4073	243	nd	768	14	nd	102
1930	2-phenylethyl butanoate	0.3	1	nd	6	2	nd	7	2	nd	9
1946	ethyl tridecanoate	3.9*	12	nd	43	5	nd	42	nd	nd	nd
1990	methyl tetradecanoate	10.7*	45	nd	163	8	nd	36	nd	nd	nd
2011	hexyl decanoate	2.3	11	nd	79	nd	nd	nd	nd	nd	nd
2031	diethyl 2-hydroxybutanedioate	22.5*	62	24	281	147	34	300	998	nd	2428
2043	ethyl tetradecanoate	8.8*	957	nd	4275	609	nd	1073	117	nd	723
2120	ethyl 3-phenylprop-2-enoate (ethyl cinnamate)	7.4*	5	nd	19	7	nd	19	nd	nd	nd
2164	2-phenylethyl hexanoate	6.9*	17	nd	49	9	nd	20	3	nd	29
2166	methyl 14-methylpentadecanoate	21.7*	120	nd	282	49	nd	91	nd	nd	nd
2232	ethyl pentadecanoate	0.7	247	nd	1848	376	nd	1620	127	nd	1281
2235	ethyl hexadecanoate	16.4*	1345	nd	2410	612	nd	1554	187	nd	1532
2280	ethyl (*E*)-hexadec-9-enoate	3.3*	74	nd	404	164	nd	764	12	nd	158
2376	2-phenylethyl octanoate	6.3*	58	nd	193	32	nd	109	6	nd	58
2395	4-ethoxy-4-oxobutanoic acid (monoethyl succinate)	33.3*	242	82	764	619	268	1272	1182	461	2164
2439	2-phenylethyl nonanoate	9.3*	nd	nd	nd	nd	nd	nd	5	nd	14
2442	ethyl octadecanoate	0.1	41	nd	306	35	nd	209	24	nd	440
	**Carbonyls**										
1386	nonan-2-one	0.3	11	nd	129	22	nd	119	11	nd	71
1390	nonanal	0.2	9	nd	180	2	nd	16	6	nd	22
1495	decanal	0.2	7	nd	53	12	nd	88	10	nd	37
1520	(*E*)-6-methylhept-2-en-4-one	46.6*	nd	nd	nd	295	132	678	nd	nd	nd
1520	benzaldehyde	17.2*	191	101	427	nd	nd	nd	291	34	551
1624	3-methylbenzaldehyde	4.0*	nd	nd	nd	1	nd	5	2	nd	12
1630	2-phenylacetaldehyde	21.4*	89	8	269	208	nd	355	277	162	469
1642	4-methylbenzaldehyde	2.2	3	nd	31	49	nd	389	4	nd	19
1660	1-phenylethanone	10.9*	1	nd	5	1	nd	7	35	nd	144
1709	dodecanal	2.2	112	nd	559	74	nd	206	36	nd	246
1780	2,4-dimethylbenzaldehyde	0.8	189	nd	906	349	nd	1050	189	nd	897
	**Furanoids, pyranoids and lactones**										
1454	furan-3-carbaldehyde	0.5	21	nd	98	34	nd	105	29	nd	163
1457	furan-2-carbaldehyde (furfural)	18.6*	42	nd	291	123	13	199	1208	98	3520
1529	1-(furan-2-yl) propan-1-one	6.6*	nd	nd	nd	nd	nd	nd	19	nd	107
1567	5-methylfuran-2-carbaldehyde (5-methylfurfural)	35.9*	7	2	34	30	9	74	271	31	623
1570	1-(5-methylfuran-2-yl) ethenone (nutty furan)	15.2*	nd	nd	nd	1	nd	9	40	nd	129
1599	ethyl furan-2-carboxylate (ethyl 2-furoate)	6.6*	130	44	571	139	59	429	393	51	1055
1656	furan-2-ylmethanol	11.6*	nd	nd	nd	1	nd	6	11	nd	49
1745	*2H*-furan-5-one	0.5	20	12	41	21	10	33	28	nd	147
1794	5-ethoxyoxolan-2-one (*γ*-ehoxybutyrolactone)	12.3*	nd	nd	nd	nd	nd	nd	4	nd	11
1911	5-butyloxolan-2-one (*γ*-octalactone)	39.7*	8	nd	30	199	52	495	13	nd	66
1964	(*4R*,*5R*)-5-butyl-4-methyloxolan-2-one (*cis*-whiskey lactone)	27.2*	nd	nd	nd	189	nd	495	519	148	1212
1978	furan-2,5-dicarbaldehyde	10.9*	19	10	43	41	10	98	87	31	314
2018	5-pentyloxolan-2-one (*γ*-nonalactone)	0.6	104	nd	329	73	nd	438	133	nd	447
2030	3-hydroxy-4,4-dimethyloxolan-2-one (pantolactone)	11.2*	1	nd	14	1	nd	5	12	nd	32
2096	5-acetyloxolan-2-one (solerone)	15.0*	nd	nd	nd	nd	nd	nd	26	nd	87
2110	5-hexyloxolan-2-one (*γ*-decalactone)	8.4*	4	nd	40	nd	nd	nd	33	nd	85
2157	5-oxooxolane-2-carboxylic acid (2-hydroxyglutaric acid *γ* -lactone)	6.3*	4	nd	38	159	128	195	74	nd	540
2168	ethyl 5-oxooxolane-2-carboxylate	6.0*	81	nd	185	159	128	195	235	nd	749
2393	2,3-dihydro-1-benzofuran (coumarin)	3.8*	89	nd	506	31	nd	111	23	nd	69
2492	5-(hydroxymethyl) furan-2-carbaldehyde	4.2*	34	10	109	70	9	185	208	36	1247
2556	furan-2-carboxylic acid	11.5*	nd	nd	nd	nd	nd	nd	125	nd	360
1616	oxolan-2-one (*γ*-butyrolactone)	2.7	94	40	334	131	76	270	138	46	248
1978	4-methyl-2,3-dihydropyran-6-one	10.5*	nd	nd	nd	nd	nd	nd	17	nd	47
2324	*3H*-2-benzofuran-1-one (phtalolactone)	2.7	1	nd	6	2	nd	8	nd	nd	nd

RI	Compound	FR	Varietal wines			Tokajské samorodné suché			Tokaj selections		
			avg	min	max	avg	min	max	avg	min	max
1956	6-propyloxan-2-one (δ-octalactone)	1.0	1	nd	3	nd	nd	nd	2	nd	13
2175	6-pentylpyran-2-one	0.5	8	nd	151	1	nd	6	2	nd	26
2246	(*R*)-5,6-dihydro-6-pentyl-*2H*-pyran-2-one (massoia lactone)	8.1*	nd	nd	nd	nd	nd	nd	15	nd	53
2266	3,5-dihydroxy-6-methyl-2,3-dihydropyran-4-one (hydroxydihydromaltol)	1.7	nd	nd	7	1	nd	6	16	nd	138
	**Terpenoids**										
1332	(*4E,6Z*)-2,6-dimethylocta-2,4,6-triene (neo-allo ocimene)	3.2	113	nd	660	4	nd	31	nd	nd	nd
1445	6-methylhept-5-en-2-ol (sulcatol)	2.3	4	nd	39	nd	nd	nd	nd	nd	nd
1460	(*3E,5E*)-2,6-dimethylocta-1,3,5,7-tetraene (cosmene)	2.8	119	nd	844	20	nd	109	21	nd	54
1479	4-methyl-2-(2-methylprop-1-enyl)-3,6-dihydro-*2H*-pyran (nerol oxide)	0.8	73	nd	403	29	nd	120	56	nd	144
1539	3,7-dimethylocta-1,6-dien-3-ol (linalool)	6.6*	159	nd	533	43	nd	165	12	nd	50
1580	2-(4-methylcyclohex-3-en-1-yl) propanal (carvomenthenal)	3.4*	18	nd	102	9	nd	42	36	nd	71
1594	4-methyl-1-propan-2-ylcyclohex-3-en-1-ol (4-terpinenol)	0.6	2	nd	22	nd	nd	nd	2	nd	18
1605	(*5E*)-3,7-dimethylocta-1,5,7-trien-3-ol (hotrienol)	2.7	116	6	454	51	18	172	47	nd	93
1617	2,6,6-trimethylcyclohexa-1,3-diene-1-carbaldehyde (*β*-safranal)	10.4*	nd	nd	nd	nd	nd	nd	11	nd	37
1630	2-methyl-6-methylideneoct-7-en-2-ol (myrcenol)	0.8	2	nd	18	nd	nd	nd	2	nd	15
1640	(*5Z*)-2,6-dimethylocta-5,7-dien-2-ol (*cis*-ocimenol)	7.1*	6	nd	39	4	nd	22	21	nd	47
1648	3,7-dimethyloct-6-enyl acetate (citronellyl acetate)	1.8	11	nd	123	nd	nd	nd	nd	nd	nd
1657	methyl (*2Z*)-3,7-dimethylocta-2,6-dienoate (methyl nerate)	54.1*	nd	nd	nd	110	nd	237	nd	nd	nd
1657	2-(4-methylcyclohex-3-en-1-yl) propan-2-ol (*α*-terpineol)	6.5*	133	7	523	nd	nd	nd	207	13	403
1670	3,7-dimethylocta-2,6-dienal (citral)	3.9*	3	nd	17	nd	nd	nd	nd	nd	nd
1676	(2,6,6-trimethylcyclohex-2-en-1-yl) methanol (*α*-cyclogeraniol)	4.1*	5	nd	32	nd	nd	nd	nd	nd	nd
1677	methyl (*2E*)-3,7-dimethylocta-2,6-dienoate (methyl geranate)	3.6*	2	nd	10	nd	nd	nd	nd	nd	nd
1712	(*3R,6S*)-6-ethenyl-2,2,6-trimethyloxan-3-ol (linalool oxide *cis* pyranoid)	2.8	9	nd	83	nd	nd	nd	nd	nd	nd
1740	(*3E,6E*)-3,7,11-trimethyldodeca-1,3,6,10-tetraene (*α*-farnesene)	11.8*	23	nd	70	9	nd	46	nd	nd	nd
1755	(*3S*)-3,7-dimethyloct-6-en-1-ol (*β*-citronellol)	7.3*	86	7	346	68	12	202	5	nd	45
1762	ethyl (*2E*)-3,7-dimethylocta-2,6-dienoate (ethyl geranate)	2.1	23	nd	171	3	nd	24	4	nd	53
1766	3,7-dimethyloct-7-en-1-ol (*α*-citronellol)	3.2	7	nd	39	1	nd	8	nd	nd	nd
1773	(*E*)-1-(2,6,6-trimethylcyclohexa-1,3-dien-1-yl) but-2-en-1-one (*β*-damascenone)	4.2*	209	49	545	402	12	889	236	26	508
1780	(*2Z*)-3,7-dimethylocta-2,6-dien-1-ol (nerol)	5.5*	48	nd	201	2	nd	18	nd	nd	nd
1901	(*1S*)-4,7-dimethyl-1-propan-2-yl-1,2-dihydronaphthalene (*α*-calacorene)	0.0	33	nd	156	36	nd	144	34	nd	105
2030	3,7,11-trimethyldodeca-1,6,10-trien-3-ol (nerolidol)	3.1	8	nd	46	2	nd	8	nd	nd	nd
2037	3,8-dimethyl-5-propan-2-yl-1,2-dihydronaphthalene (*α*-corocalene)	0.4	11	nd	35	16	nd	104	9	nd	33
2315	(*2E*)-3,7-dimethylocta-2,6-dienoic acid (geranic acid)	2.6	36	nd	237	4	nd	31	nd	nd	nd
N	oxane-2,6-dione (glutaric anhydride)	15.6*	nd	nd	nd	nd	nd	nd	33	nd	100
1582	(*3E*)-6-methylhepta-3,5-dien-2-one	3.3*	1	nd	6	nd	nd	nd	3	nd	10
1560	1,1,6-trimethyl-1,2,3,4-tetrahydronaphthalene (*α*-ionene)	3.0	9	nd	79	2	nd	14	29	nd	132
2000	1,7-dimethylnaphthalene	0.8	8	nd	59	nd	nd	nd	8	nd	69
2182	1,6,7-trimethylnaphthalene	8.8*	5	nd	20	9	nd	25	nd	nd	nd
2200	1,6-dimethyl-4-propan-2-ylnaphthalene (cadalene)	0.5	46	17	135	62	24	202	51	nd	132

RI	Compound	FR	Varietal wines avg	min	max	Tokajské samorodné suché avg	min	max	Tokaj selections avg	min	max
1836	(*1R,8aS*)-1,8a-dimethyl-7-propan-2-ylidene-1,2,6,8-tetrahydronaphthalene (*β*-vetivenene)	2.6	10	nd	42	nd	nd	nd	4	nd	33
2450	2-[(*1E*)-1,3-Butadien-1-yl]-1,3,4-trimethylbenzene (TPB)	46.1*	nd	nd	nd	nd	nd	nd	246	47	491
1686	1,1,6-trimethyl-*2H*-naphthalene (TDN)	21.2*	141	nd	812	576	nd	1120	1375	217	3081
	**Volatile phenols**										
1860	2-methoxyphenol (gauiacol)	29.6*	nd	nd	nd	2	nd	5	11	nd	24
1992	phenol	9.4*	33	15	117	158	22	534	nd	nd	nd
2183	4-ethylphenol	5.9*	7	nd	44	92	nd	622	178	nd	641
2191	2-methoxy-4-prop-2-enylphenol (eugenol)	30.6*	nd	nd	nd	26	nd	40	24	nd	56
2192	4-ethenyl-2-methoxyphenol (4-vinylguaiacol)	3.5*	60	nd	281	11	nd	42	32	nd	129
2693	4-(1,1-dimethylpropyl) phenol	12.1*	nd	nd	nd	nd	nd	3	761	nd	2044
	**Volatile sulphur compounds**										
1534	2-methylthiolan-3-one (blackberry tiophenone)	9.2*	5	nd	14	3	nd	14	nd	nd	nd
1557	ethyl 3-methylsulfanylpropanoate	7.5*	2	nd	8	4	nd	8	13	nd	39
1705	3-methylsulfanylpropan-1-ol (methionol)	8.7*	27	13	65	37	17	54	60	23	135
1948	1,3-benzothiazole	8.5*	1	nd	15	nd	nd	nd	23	nd	82

Legend: Relative concentrations are expressed as equivalents of benzophenone in $\mu g.l^{-1}$. RI - experimental value of retention index; avg – average value of relative concentration; min – minimal determined concentration; max – maximal determined concentration; *FR* – Fischer ratio; nd – not detected; * indicates $p \leq 0.05$
